# Safety, efficacy, OCT-assessed vascular healing and angiographic outcomes of polymer-free versus biodegradable-polymer drug-eluting stents in patients undergoing percutaneous coronary intervention: a systematic review and meta-analysis

**DOI:** 10.1186/s40001-026-03999-5

**Published:** 2026-02-20

**Authors:** Merna M. Abouelenien, Mennatullah E. Mekky, Zeyad Bakry, Yazan Sawafta

**Affiliations:** Faculty of Medicine, Alexandria University, Alexandria, Egypt

## Abstract

**Background:**

Biodegradable polymer (BP) and polymer-free (PF) drug eluting stents were designed to reduce late adverse events linked to durable-polymer coatings. However, concerns remain regarding the efficacy of PF–DES particularly due to variations in drug-release mechanisms.

**Methods:**

RCTs comparing PF–DES and BP–DES in patients undergoing percutaneous coronary interventions were included. Primary endpoints were Target lesion revascularization (TLR), Target vessel revascularization (TVR), definite stent thrombosis, Cardiac death (CD), angiographic outcomes and optical coherence tomography (OCT) findings. Risk of bias was assessed using Cochrane’s ROB 2.0 and meta-analysis performed using RevMan 5.4.

**Results:**

Ten RCTs including 9020 patients (4043 PF–DES; 4977 BP–DES) were included. At 12 months, PF–DES showed a significantly higher TLR rate (2.08% vs. 1.36%; RR 1.55, 95% CI 1.087–2.21; *P* = 0.02), persisting at 24 months (RR = 2.01, 95% CI 1.46–2.77, *P* < 0.0001). At 1–3 months, OCT-derived analyses demonstrated no statistically significant differences in uncovered struts or neointimal thickness between stent types, while findings related to malapposed struts were heterogenous and dependant on sensitivity analyses.

Angiographically, PF–DES showed greater in-stent late lumen loss at 6–8 months ([MD]: 0.24 mm, 95% CI 0.17–0.30, *P* < 0.00001), whereas minimal lumen diameter was comparable.

However, no significant differences were found in target vessel revascularization (TVR), stent thrombosis, myocardial infarction, or mortality at 1- or 2-year follow-up.

**Conclusions:**

PF–DES were associated with comparable safety to BP–DES; however, it exhibited higher rates of target lesion revascularization and greater late lumen loss were observed. OCT-derived findings were heterogenous and should be interpreted cautiously. Further RCTs with standardized imaging protocols and longer follow-up are warranted.

## Introduction

Percutaneous coronary intervention (PCI) with Drug Eluting Stents (DES) is one of the main treatments for coronary artery diseases, helping to decrease the risk of in-stent restenosis and reducing the need for further procedures on the same blood vessel (target vessel revascularization) [1, 2].

Drug-eluting stents (DES) have had a major positive impact on patients’ outcomes after percutaneous coronary intervention (PCI) compared to bare-metal stents (BMS); as they reduce the need for revascularization and are linked to a lower risk of major adverse cardiovascular events (MACE) but with no difference in mortality [3, 4].

However, growing concerns have emerged about late adverse events after durable drug-eluting stent (DES) implantation [5]. Leftover polymer in the coronary artery may trigger a prolonged inflammatory response in the vessel wall, which can increase the risk of late stent blockage due to clot formation and promote excessive tissue growth inside the stent (neointimal overgrowth) [6, 7].

In the hope of further reducing the rate of late adverse events after stenting, a biodegradable polymer was introduced. This polymer controls the release of the drug coating it during a suitable and specific period, and after that erodes leaving no polymer residue on the stent [4, 8].

In addition, an entirely polymer-free DES was created as a newer advancement in stent technology, designed to bypass polymers entirely, employing drug-impregnated surfaces or micro-reservoirs to avoid inflammatory polymer remnants [9, 10].

A landmark 2018 meta-analysis by Nogic et al. demonstrated significantly lower rates of target lesion revascularization (TLR) (OR 0.48) and late lumen loss (mean difference–0.20 mm) with BP–DES while showing no significant differences in all-cause death, myocardial infarction (MI), or stent thrombosis [9].

Recent randomized trial by Han et al. compared ultrathin PF Coroflex (70–80 µm) with ultrathin BP Orsiro stents showing significantly higher target lesion failure with PF Coroflex, which suggests that even with ultrathin platforms, PF–DES may fall back in long-term efficacy [11].

In contrast to Han et al., SORT OUT IX comparing PF BioFreedom with BP Orsiro showed no statistically significant difference in TLF at 2 years, however, showed nearly double the TLR rate with PF–DES (5.1% vs. 2.6%; RR 1.98), especially in the first year (3.5% vs. 1.3%; RR 2.77) indicating early effectiveness concerns with polymer-free technology [12].

While insightful, most of these studies used earlier-generation devices and did not assess OCT-defined vascular healing, which includes strut coverage, neointimal thickness, and malapposition, key markers predicting thrombosis risk and endothelial recovery.

Optical coherence tomography (OCT) has now become essential in evaluating in vivo healing. Recent randomized OCT trials [9, 13–15] have compared PF–DES and BP–DES for early strut coverage and late healing patterns. Fernández et al. a multicenter OCT study, found that over 80% of stent struts remained uncovered at 1-month post-implantation across polymer-free, biodegradable-polymer, and durable-polymer DES (80.2%, 88.1%, and 82.5%, respectively; *P* = 0.209). However, by 6 months, strut coverage significantly improved to 97%, 95%, and 93.7%, with no significant differences among groups (*P* = 0.172) [14]. This demonstrates that early vascular healing is equally suboptimal in PF–DES and BP–DES, with both platforms achieving robust mid-term endothelialization.

Similarly, angiographic outcomes, such as minimal lumen diameter (MLD) and LLL, although important indicators of neointimal proliferation, have been inconsistently reported, which remain important indicators of in-stent restenosis and neointimal thickness. For instance, **ISAR-TEST-3** trial reported significantly higher LLL with polymer-free DES at 6–8 months, though this difference was no longer evident at 2 years due to late “luminal creep” observed in BP and PP DES, but not in PF DES [16]. Similarly, **Fernández et al.** evaluated LLL and MLD at 1 and 6 months and found no significant inter-group differences, though PF stents showed slightly more neointimal hyperplasia at 6 months [14].

As a result, due to the conflicting results across different follow-up times, the introduction of new data and the absence of a comprehensive meta-analysis that integrates clinical, angiographic, and OCT-derived vascular healing outcomes to directly compare PF–DES with modern BP–DES across only randomized controlled trials.

Our aim is to compare the efficacy and safety of biodegradable polymer-coated drug-eluting stents versus polymer-free stents by analyzing clinical and angiographic outcomes and using optical coherence tomography (OCT) to assess short-term arterial healing. By integrating established clinical endpoints with mechanistic OCT data and angiography, this work represents the most comprehensive examination to date, clarifying whether PF–DES truly offer clinical or vascular-healing advantages over advanced BP–DES.

## Methods

This Systematic review and meta-analysis of ten RCTs was performed according to the Preferred Reporting Items for Systematic Reviews and Meta-Analyses (the PRISMA 2020 update) [17] and followed the recommendations of the Cochrane handbook for Systematic Reviews of Interventions [18]. The protocol was registered in the International Prospective Register of Systematic Reviews (PROSPERO) with the following registration ID: CRD420251119355. This meta-analysis was conducted at the study level, as individual patient-level data were not available from the included trials.

### Literature search

We systematically searched PUBMED, Web of Science, and Scopus from Inception till January 23, 2025.

We used the following key terms in our search to ensure that all eligible studies were included in the literature search; “polymer-free” AND “Biodegradable” AND (“PCI” OR “Percutaneous coronary Intervention”) AND (“Stent” OR “Stents”).

The references’ lists of articles included in this review was further manually screened to include other eligible studies. Primary source studies published in peer-reviewed journals were eligible for inclusion when meeting the following criteria.

### Eligibility criteria

RCTs that compared Polymer Free DES and Biodegradable Polymer DES in adult patients with coronary artery diseases (CAD) undergoing percutaneous coronary interventions (PCI). We excluded cohort studies, case reports, editorials, conference abstracts and animal studies.

### Screening, data extraction and risk of bias

Initial title and abstract screening were conducted by two independent reviewers (Y.S. and Z.B.), and all disagreements were discussed to reach a consensus, otherwise, a third researcher (M.A.) was consulted to settle them.

Eligible articles initially included by the reviewers were imported for full-text review and assessed for final inclusion. Data extraction was performed independently by two reviewers (Y.S and Z.B) using pre-defined data extraction forms. Examples of data collected are study characteristics, number of patients in each group, age, sex (n), and other baseline diseases, event of Target Lesion Revascularization (TLR) and its components, number of patients with definite stent thrombosis and other secondary outcomes. Disagreements were resolved by consensus or discussion with a third reviewer (M.A).

Two Independent reviewers (M.M. and Z.B.) assessed the risk of bias in the included studies using Cochrane’s ROB 2.0 revised tool [19]. The following sets of domains were assessed (Randomization process, deviations from intended interventions, missing outcome data, measurement of the outcome and selection of the reported result) and studies were judged as ‘Low’ or ‘High’ risk of bias or can express ‘Some concerns’. Disagreements were discussed to reach a consensus.

### Study endpoints

The primary outcomes for this meta-analysis were: 12-month and 24-month target lesion revascularization (TLR), Target vessel revascularization (TVR), definite and overall stent thrombosis, Cardiac death (CD), in-stent late lumen loss at follow-up angiogram, optical coherence tomography (OCT) findings including percentage of uncovered struts, neointimal thickness and malapposed struts. Secondary endpoints were all-cause mortality, target-lesion-related myocardial infarction, and overall stent thrombosis.

### Statistical analysis

Review Manager 5.4 software was utilized for data analysis. The evaluation of clinical outcomes was conducted using relative risk ratios (RRs) accompanied by their corresponding 95% confidence intervals (CI) in case they were dichotomous. However, continuous data were presented as mean differences (MD) with 95% confidence intervals (CI). *I*^2^ statistic and *P* value were used to assess the statistical heterogeneity between outcomes of RCTs. Heterogeneity was considered significant if *P* < 0.1, in which case a sensitivity analysis was performed (leave-one-out-test). In cases where there was significant heterogeneity, we applied a random‐effects model. Conversely, when the heterogeneity was insignificant, a fixed‐effects model was used instead. To present these findings in a visually informative manner, forest plots were utilized. Results were considered statistically significant if a *P* value for hypothesis test was less than 0.05.

## Results

### Summary of enrolled studies and risk of bias

**Fig. 1:**
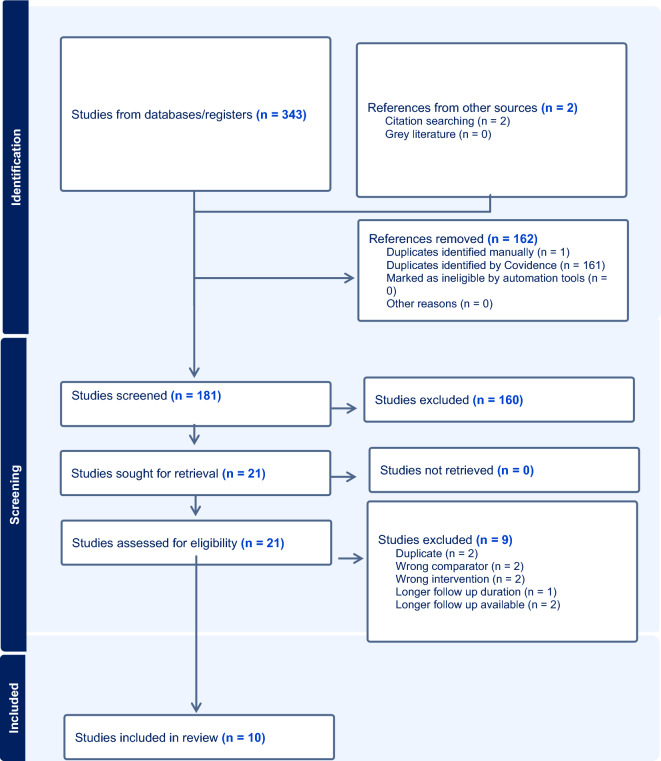
PRISMA

After a comprehensive search of the literature, 343 studies were found, 181 were eligible for title and abstract screening after duplicate removal. Of the 181 studies, 160 were irrelevant and 21 studies were eligible for full-text screening. Finally, 10 studies were included in the meta-analysis after full text screening [11–16, 20–23], as shown in the PRISMA flow chart, Fig. 1. Summary of the included studies are shown in Tables 1 and 2. The total number of patients included in the study is 9020 patients, with 4043 in the PF–DES group and 4977 in the BP–DES group. The baseline data for patients are shown in Tables 3 and 4.

**Table 1:**
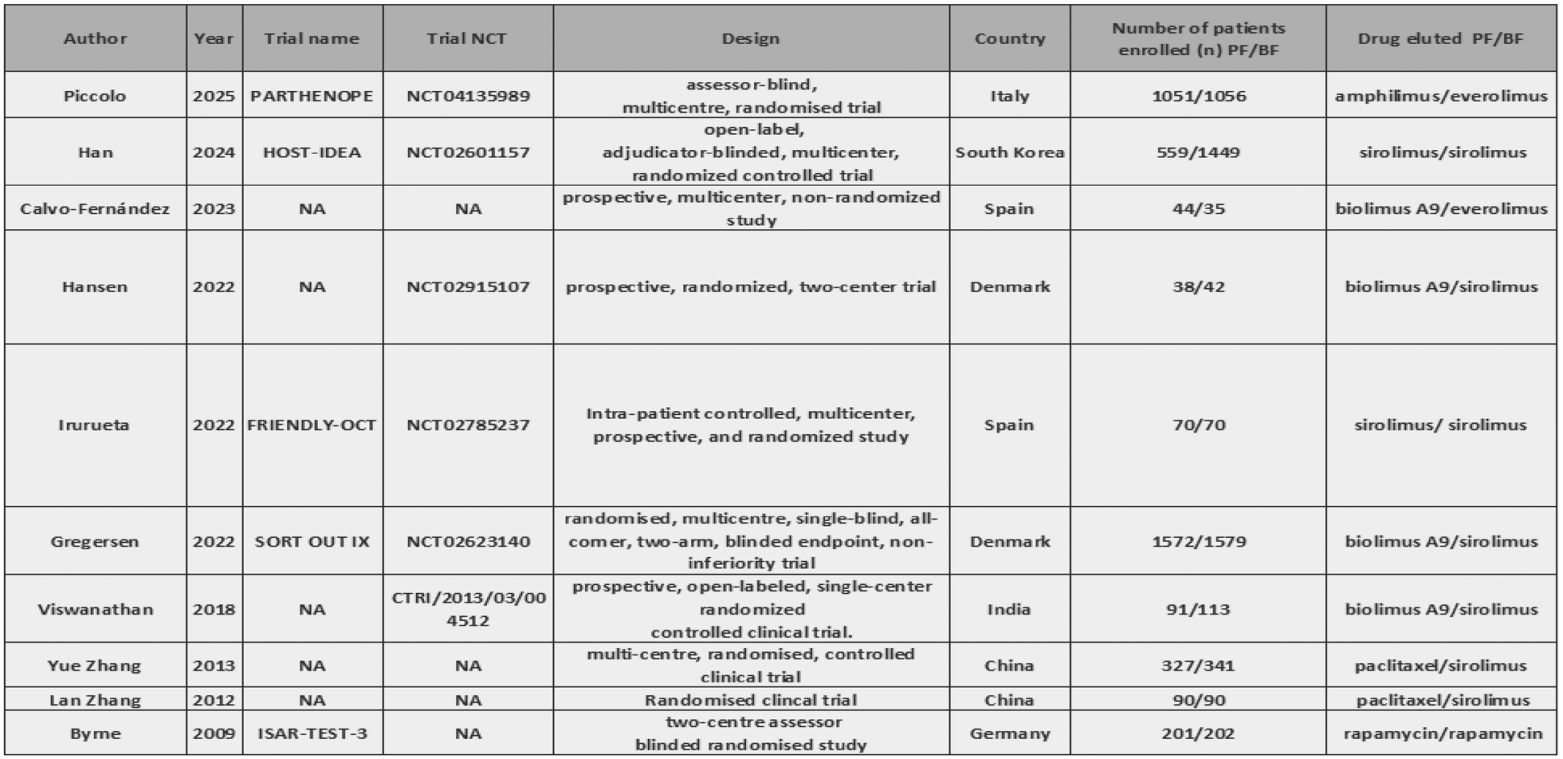
Summary of included studies A

**Table 2:**
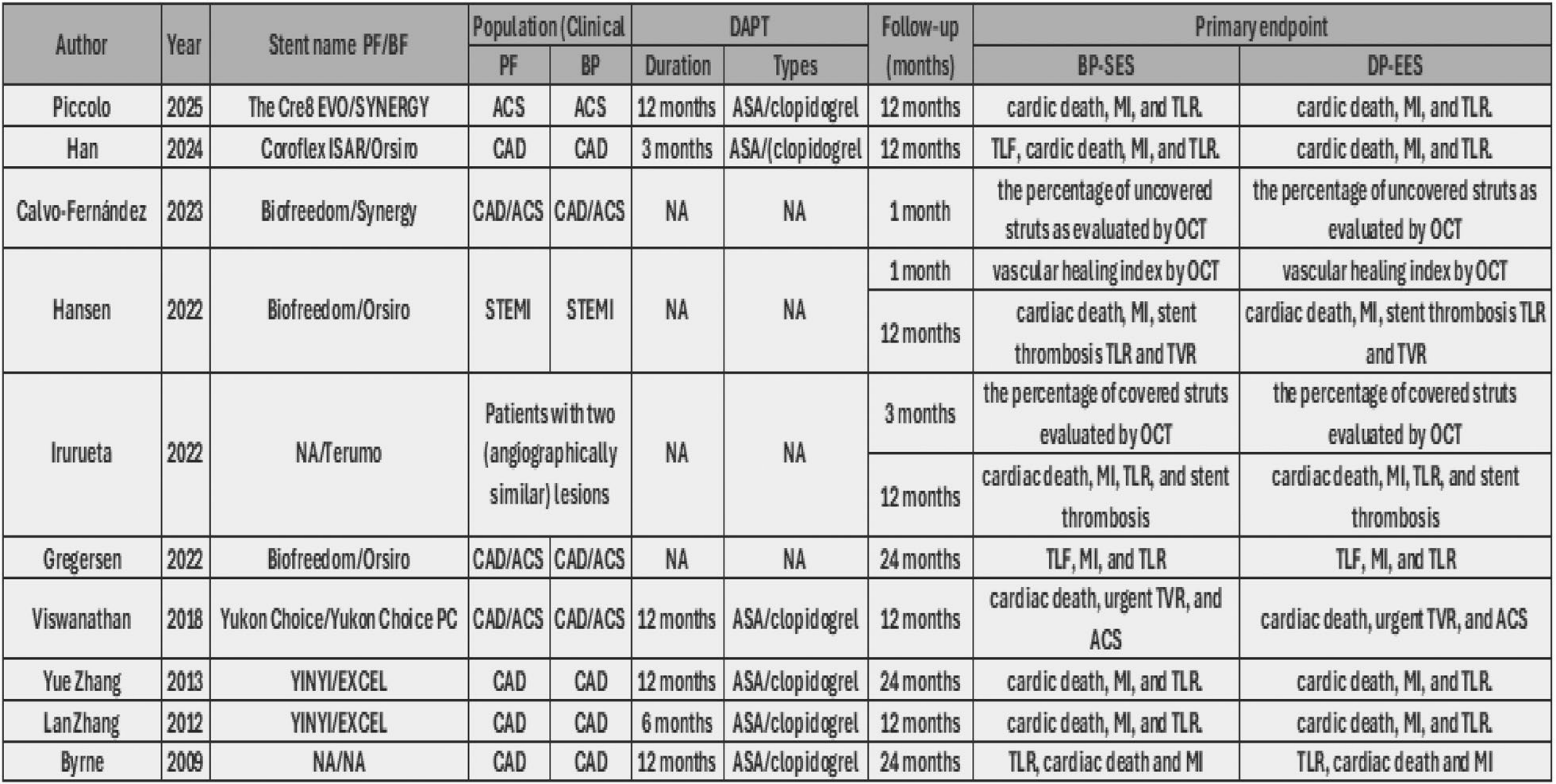
Summary of included studies B

**Table 3:**
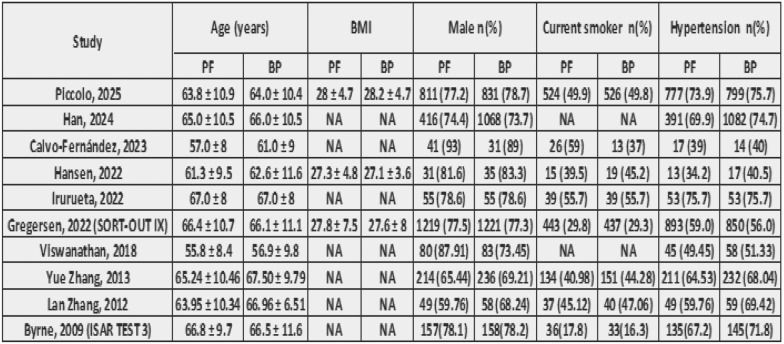
Baseline characteristics A

**Table 4:**
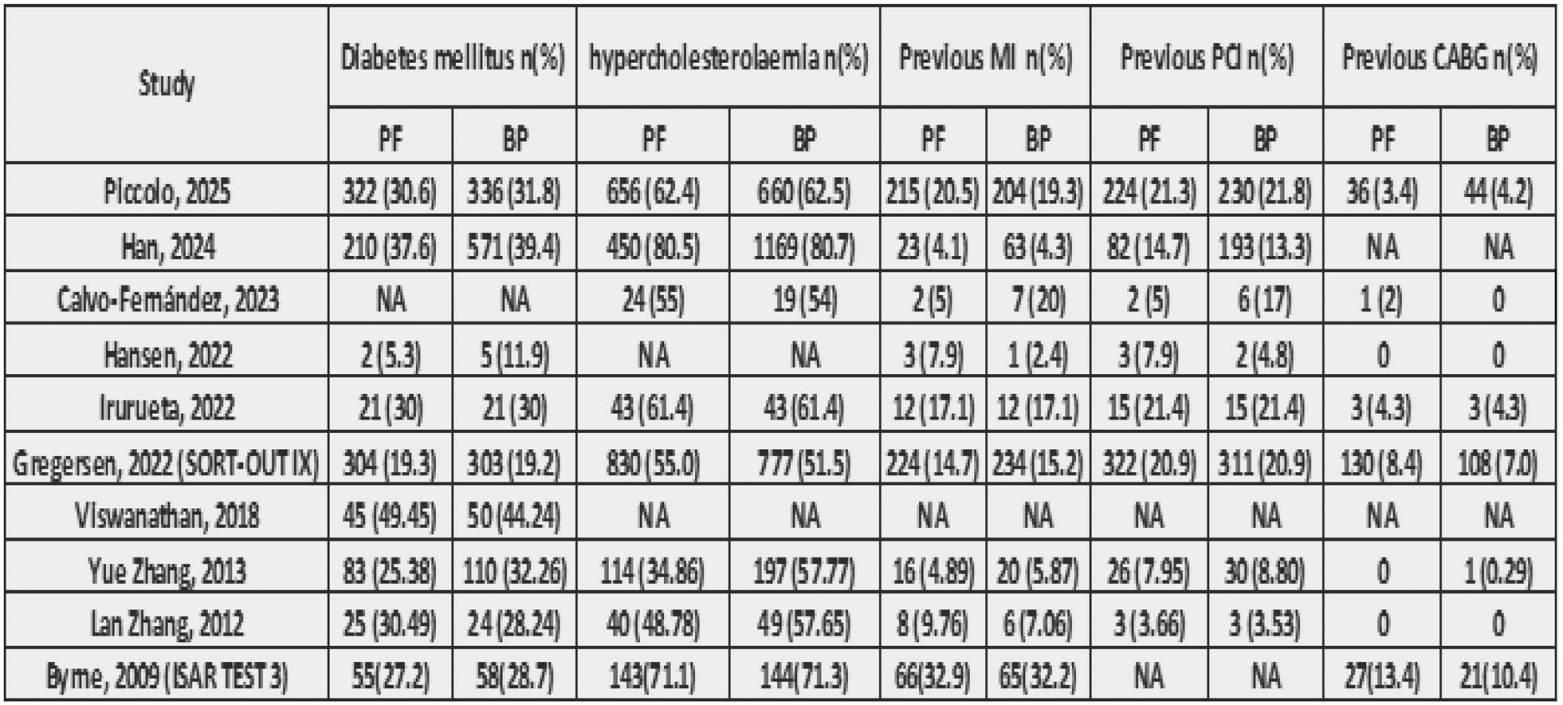
Baseline characteristics B

**Fig. 2:**
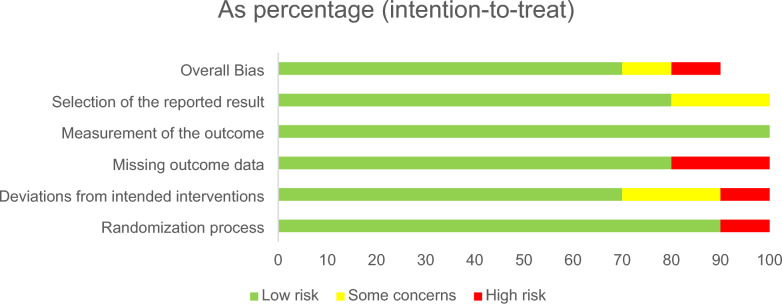
Risk of bias (ROB 2.0)

The overall risk of bias was low in most of the included studies, as 70% were judged to have low risk across all domains. However, 10% of studies were rated at high risk of bias overall, and another 10% raised some concerns. Regarding individual domains, 90% of the studies were at low risk of bias in the Randomization process domain, while the remaining 10% showed high risk of bias in such domain. The most concerning domain was missing outcome data, in which 20% of studies were at high risk, although most of the studies 80% were at low risk. All studies were at low risk for bias in the measurement of the outcome domain. Similarly, no study was found to be at high risk of bias in the selection of the reported result domain, although 20% raised some concerns. These assessments are summarized in Fig. 2.

### Outcomes

At 1-year follow-up, target lesion revascularization (TLR), definite stent thrombosis, overall stent thrombosis, and cardiac death were each reported in 6 of the 10 included studies. All-cause mortality was reported by 7 studies. Target vessel revascularization (TVR) and target lesion-related myocardial infarction (TL–MI) were each reported in 5 studies.

At 2-year follow-up, target vessel revascularization (TVR), target lesion-related myocardial infarction (TL–MI), definite stent thrombosis, and cardiac death were each reported in 2 studies. Target Lesion Revascularization (TLR), all-cause mortality and overall stent thrombosis were reported in 3 studies. The limited availability of long-term data restricted the robustness of pooled effect estimates for this timepoint.

OCT-assessed vascular healing outcomes were reported by three studies: *Hansen *et al*., 2022*, *Irurueta *et al*., 2022*, and *Fernández *et al*., 2023*. All three trials provided data on uncovered struts, malapposed struts, and neointimal thickness within 1–3-month post-PCI, enabling pooled analysis of early endothelial coverage and strut apposition between stent types.

Angiographic outcomes at 6–8 months were available from only two studies: *ISAR TEST 3, 2009*[16] and *Fernández *et al*., 2023*[14], both of which reported data on minimal lumen diameter and in-stent late lumen loss. The limited number of studies constrains the precision and generalizability of the pooled estimates for these angiographic measures.

- (1) Primary endpoints

### Clinical safety and efficacy endpoints

**Fig. 3:**
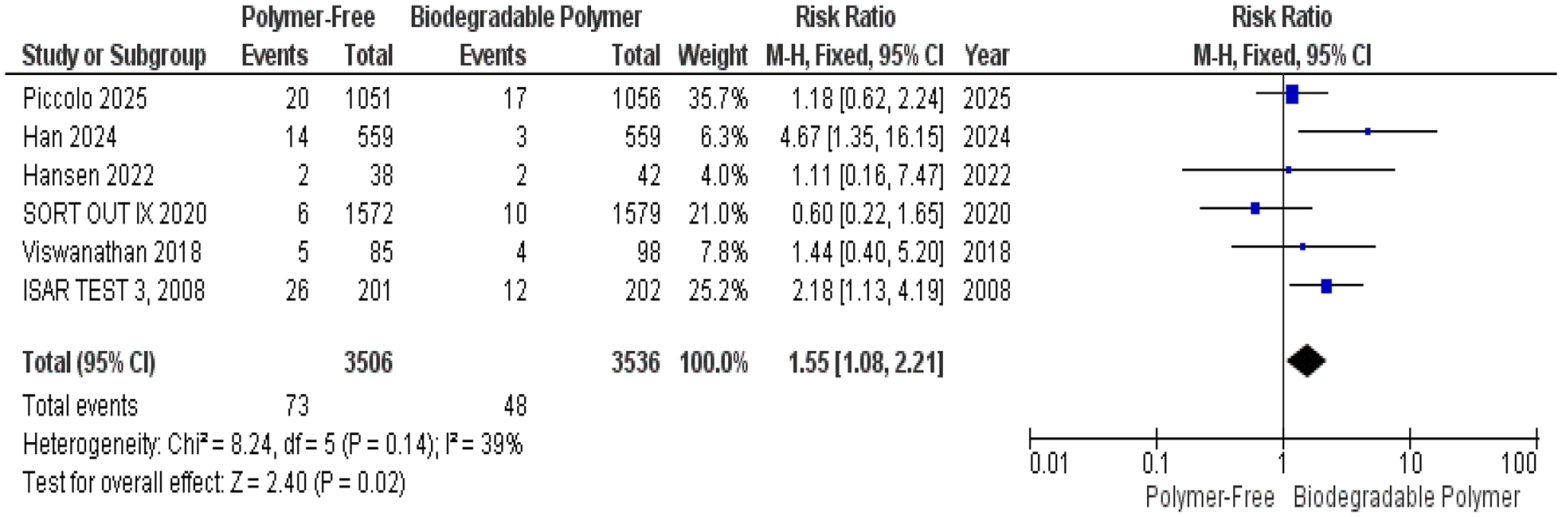
Target lesion revascularization (TLR) at 12 months

At 1-year follow-up, our analysis indicated Target Lesion Revascularization (TLR) was significantly higher in the PF–DES group compared to the BP–DES group (risk ratio [RR] = 1.55, 95% confidence interval [CI] 1.08–2.21, *P* = 0.02). Heterogeneity was not significant among the six included studies (*P* = 0.14, I^2^ = 39%), as shown in Fig. 3.

**Fig. 4:**
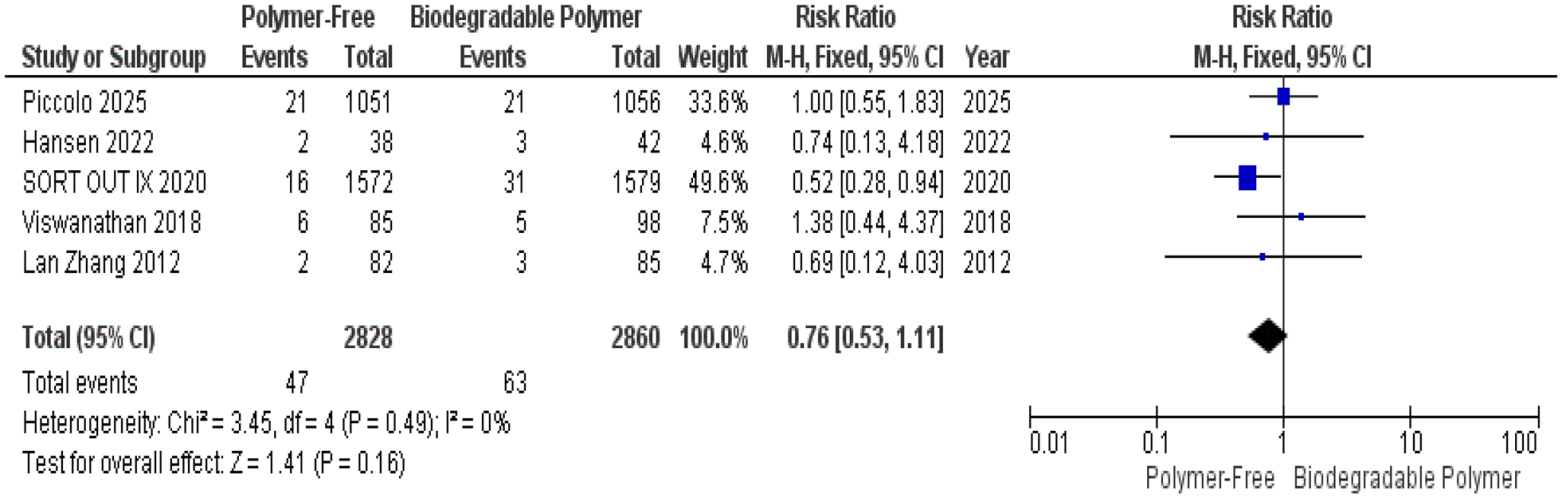
Target vessel revascularization (TVR) at 12 months

**Fig. 5:**
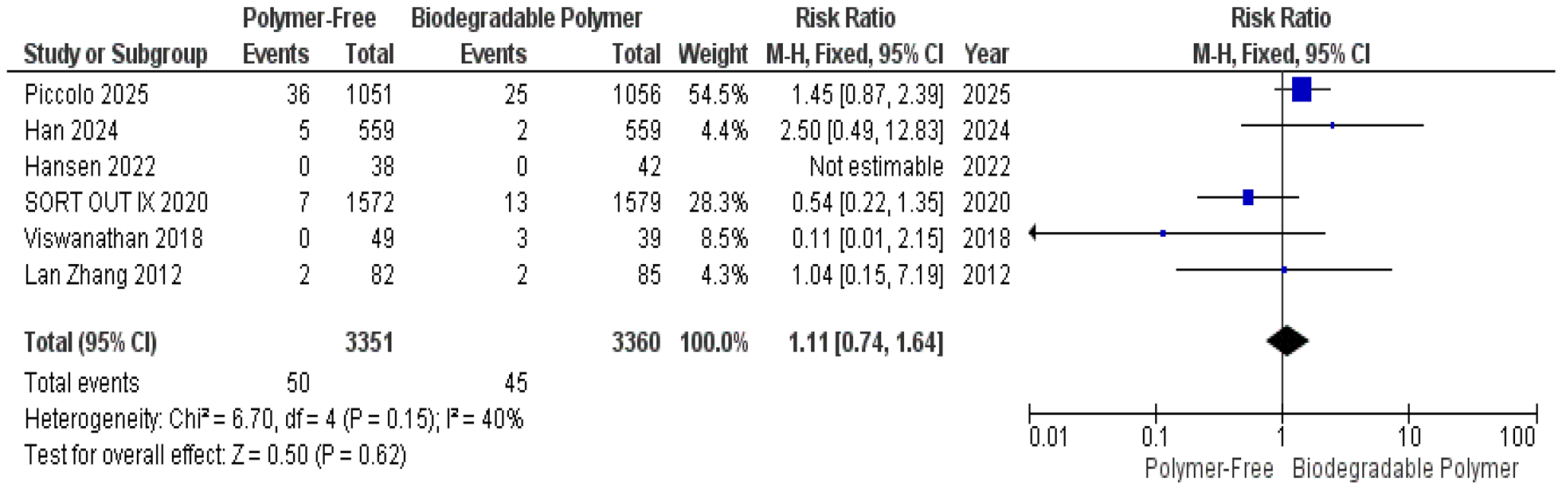
Cardiac death at 12 months

**Fig. 6:**
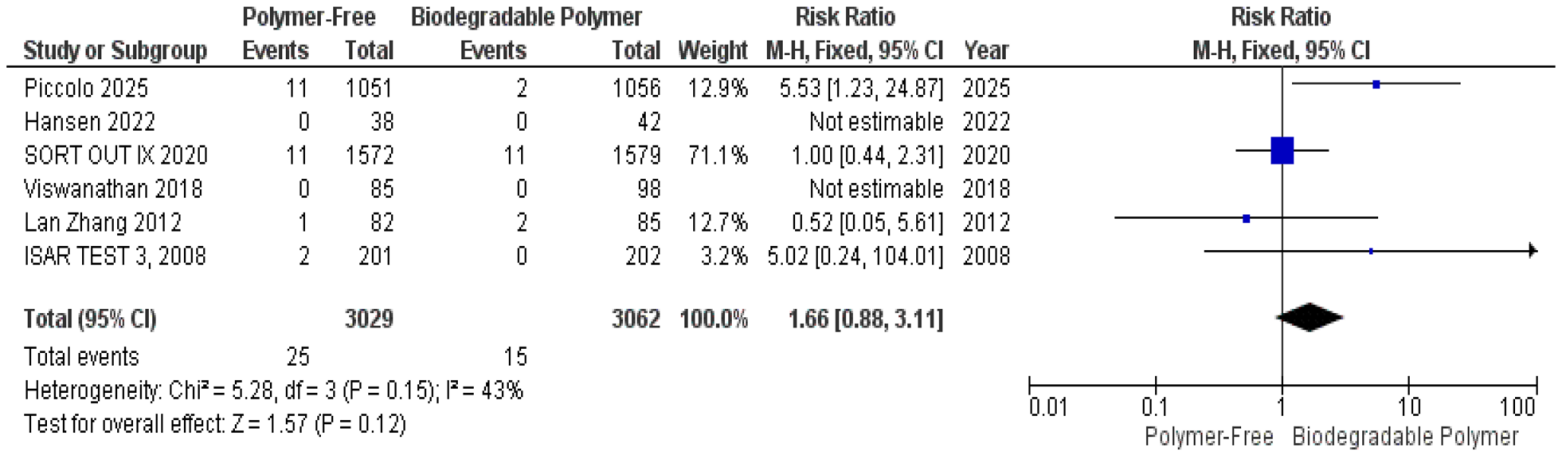
Definite stent thrombosis at 12 months

No significant differences were observed between groups for Target Vessel Revascularization (TVR) (RR = 0.76, 95% CI 0.53–1.11, *P* = 0.16) (Fig. 4), Cardiac Death (RR = 1.11, 95% CI 0.74–1.64, *P* = 0.62) (Fig. 5), and Definite Stent Thrombosis (RR = 1.66, 95% CI 0.88–3.11, *P* = 0.12) (Fig. 6).

Similarly, these outcomes showed no significant heterogeneity TVR (*P* = 0.49, I^2^ = 0%) (Fig. 4), Cardiac death (*P* = 0.15, I^2^ = 40%) (Fig. 5), definite stent thrombosis (*P* = 0.15, I^2^ = 43%) (Fig. 6).

**Fig. 7:**
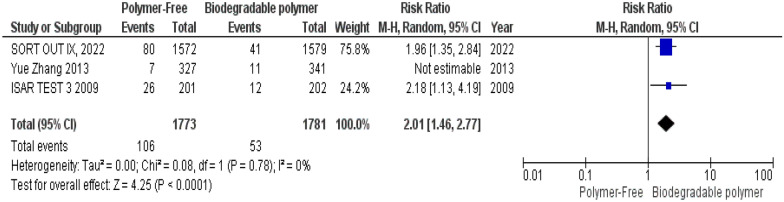
Target lesion revascularization (TLR) at 24 months

At 2-year follow-up, a statistically significant increase in TLR with polymer-free stents was observed (RR = 1.78, 95% CI 1.32–2.41, *P* = 0.0002). However, moderate statistical heterogeneity was present (I^2^ = 59%, Chi^2^ = 4.90, df = 2, *P* = 0.09). A random-effects model was then adopted as the primary analytical approach and Leave-one-out test was conducted to assess the influence of individual studies on the pooled effect and heterogeneity. Exclusion of the smallest trial (Yue Zhang et al., 2013) reduced heterogeneity to 0% and resulted in a statistically significant pooled effect (RR = 2.01, 95% CI 1.46–2.77, *P* < 0.0001), confirming the direction and significance of the overall effect (Fig. 7).

**Fig. 8:**
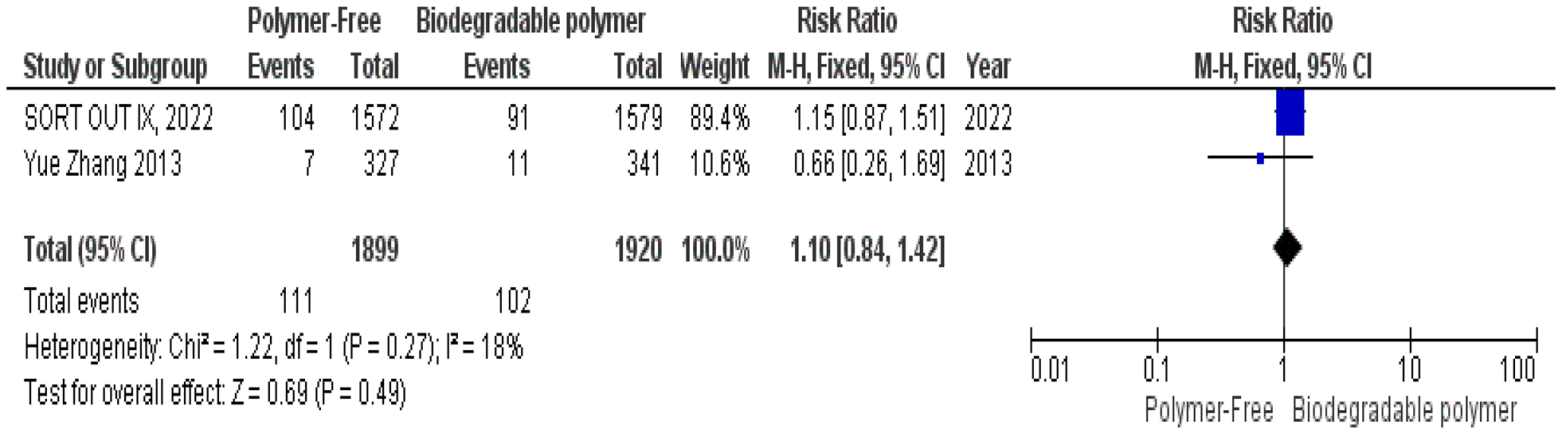
Target vessel revascularization (TVR) at 24 months

**Fig. 9:**
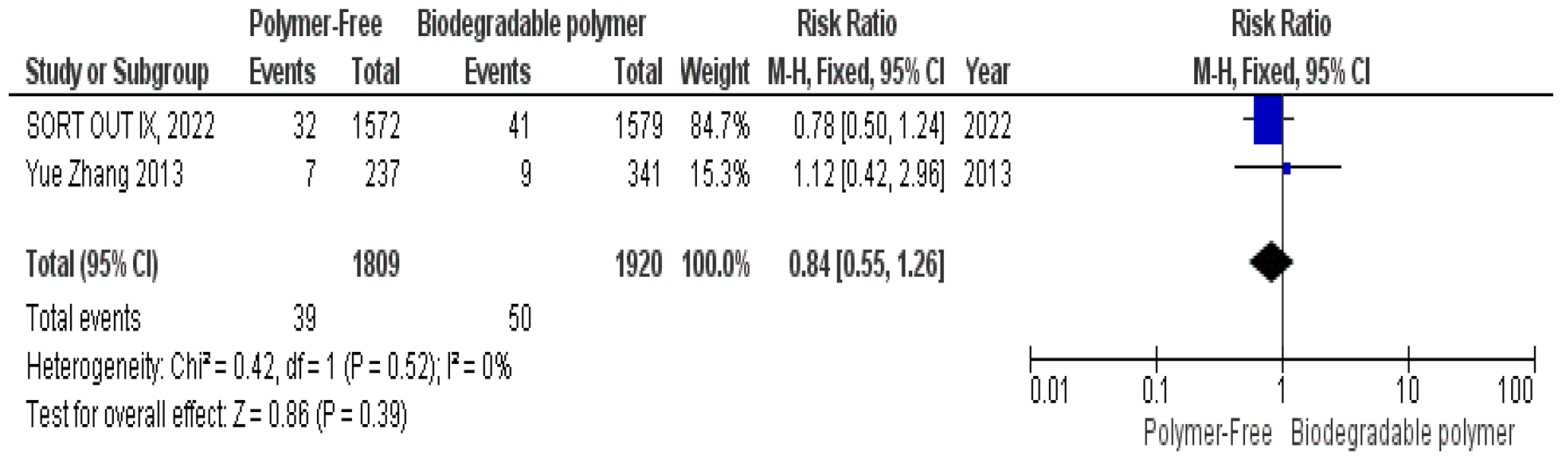
Cardiac death at 24 months

**Fig. 10:**
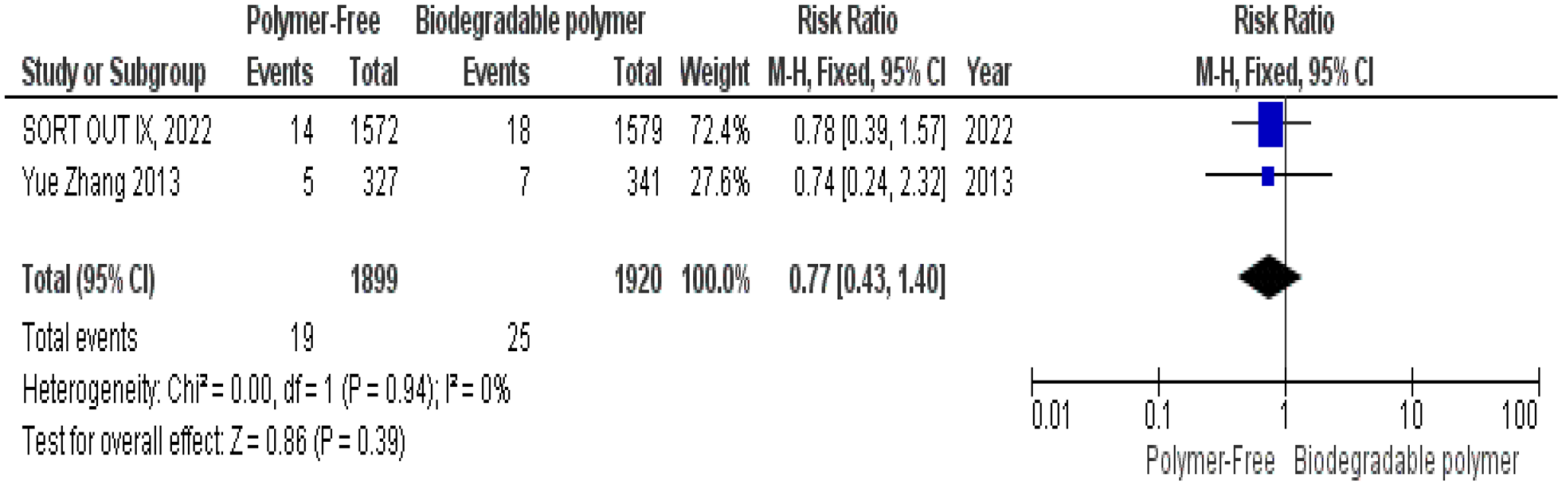
Definite stent thrombosis at 24 months

No significant differences were observed between groups regarding definite stent thrombosis (RR = 0.77, 95% CI 0.43–1.40, *P* = 0.39) (Fig. 8), cardiac death (RR = 0.84, 95% CI 0.55–1.26, *P* = 0.39) (Fig. 9) and target vessel revascularization (TVR) (RR = 1.10, 95% CI 0.84–1.42, *P* = 0.49) (Fig. 10).

Similarly, these outcomes showed no significant heterogeneity; definite stent thrombosis (*P* = 0.94, I^2^ = 0%) (Fig. 8), cardiac death (*P* = 0.52, I^2^ = 0%) (Fig. 9), and target vessel revascularization (TVR) (*P* = 0.27, I^2^ = 18%) (Fig. 10).

### Optical coherence tomography OCT outcomes at 1–3 months

OCT-assessed vascular healing parameters were reported across 3 of the included studies and analyzed using random-effects models. Given the limited number of contributing studies, variability in imaging timepoints, and substantial heterogeneity, OCT-derived findings should be interpreted cautiously.

**Fig. 11:**
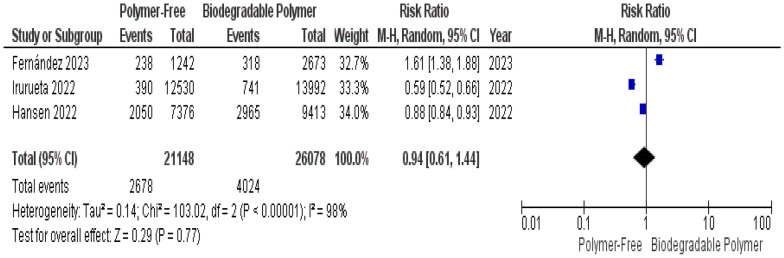
Uncovered struts at 1–3 months

Uncovered struts were reported in three studies. The pooled analysis showed no significant difference between polymer-free and biodegradable-polymer stents (RR = 0.94, 95% CI 0.61–1.44; *P* = 0.77), with substantial heterogeneity (I^2^ = 98%) (Fig. 11). Leave-one-out sensitivity analysis did not identify the source of heterogeneity or alter the overall effect direction or significance.

**Fig. 12:**
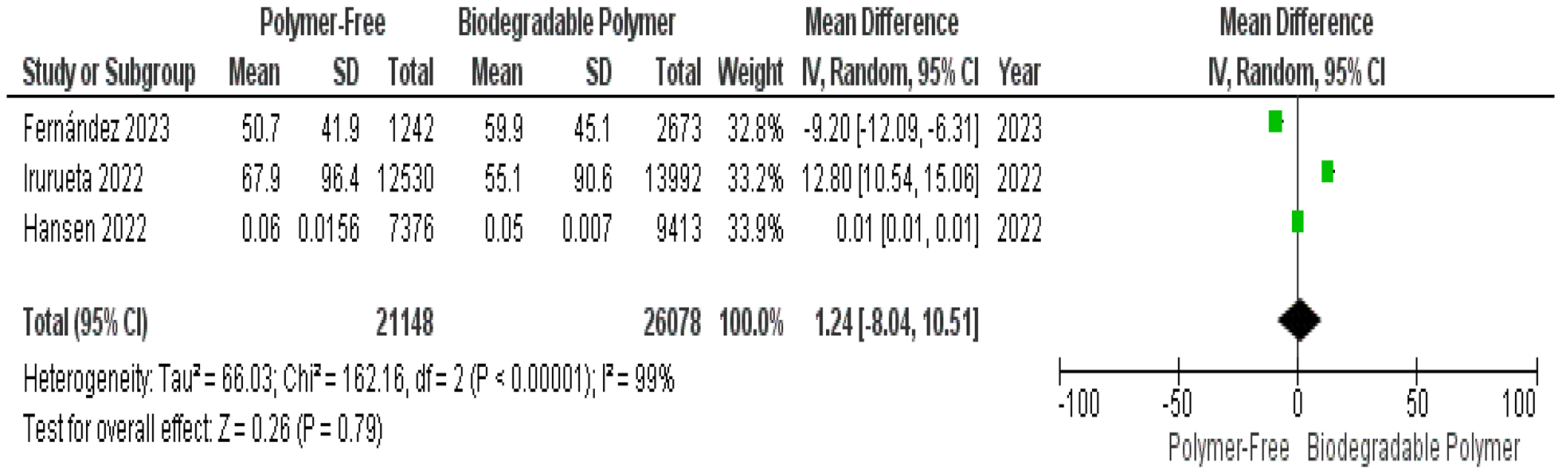
Neointimal thickness at 1–3 months

Similarly, neointimal thickness did not significantly differ between the two stent types (MD = 1.24 µm, 95% CI –8.04 to 10.51; *P* = 0.79), with considerable heterogeneity observed (I^2^ = 99%) (Fig. 12). Sensitivity analysis did not identify the source of heterogeneity or alter the overall effect direction or significance.

**Fig. 13:**
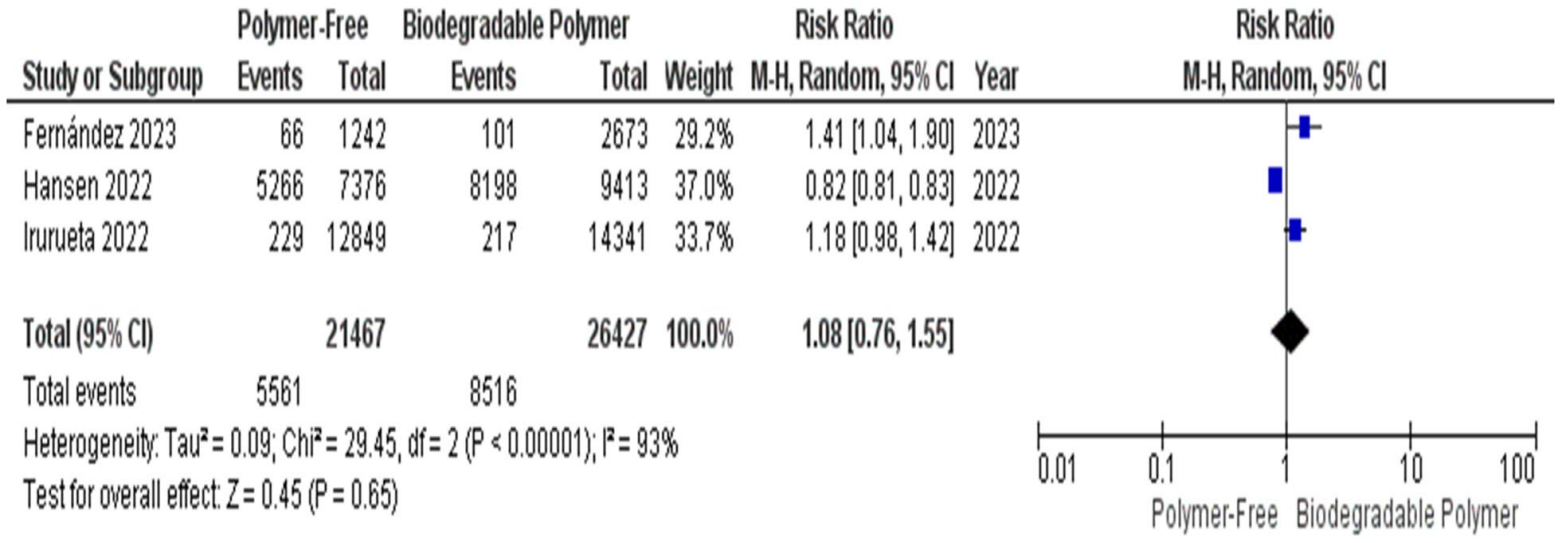
Malapposed struts at 1–3 months

For malapposed struts, the initial random-effects analysis showed no significant difference between groups (RR = 1.08, 95% CI 0.76–1.55; *P* = 0.65) and revealed substantial heterogeneity (I^2^ = 93%) (Fig. 13). Sensitivity analysis indicated that the exclusion of a heterogeneity-driving study resulted a statistically significant pooled effect estimate (RR = 1.24, 95% CI 1.06–1.45; *P* = 0.008) with reduced heterogeneity (I^2^ = 0%); however, this finding was dependent on study inclusion and should, therefore, be interpreted with caution. Accordingly, the initial analysis including all available studies is presented without implying a difference in malapposition between stent types.

- (2) Secondary endpoints

### Clinical safety and efficacy endpoints

**Fig. 14:**
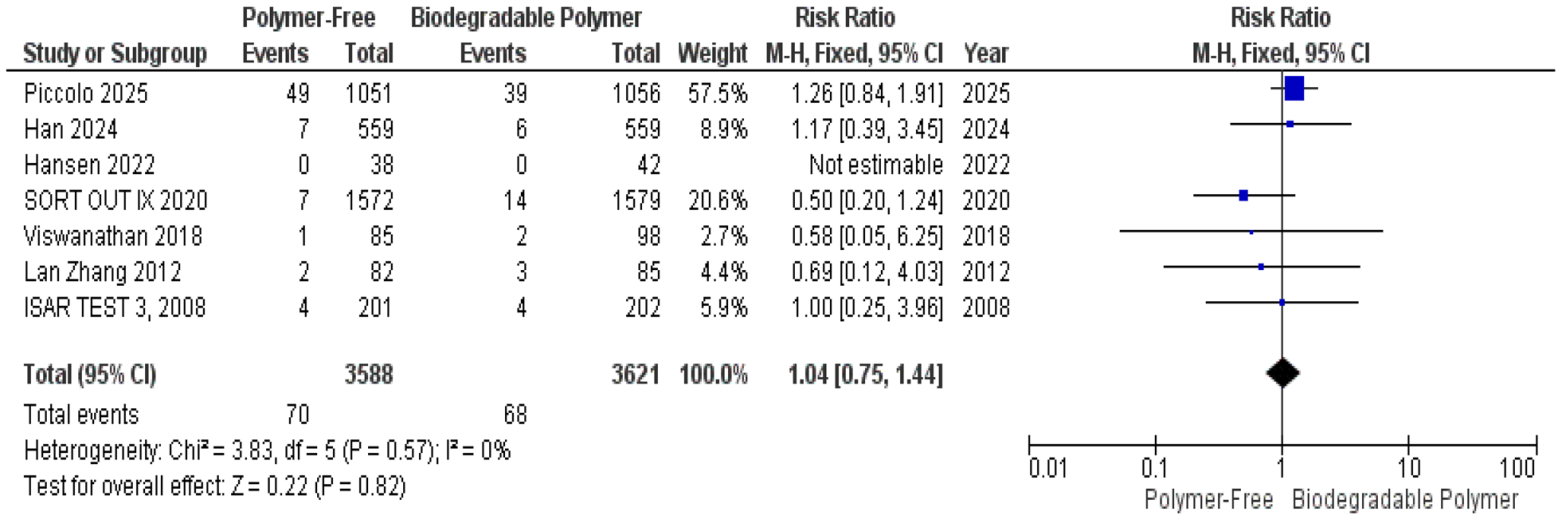
All-cause mortality at 12 months

**Fig. 15:**
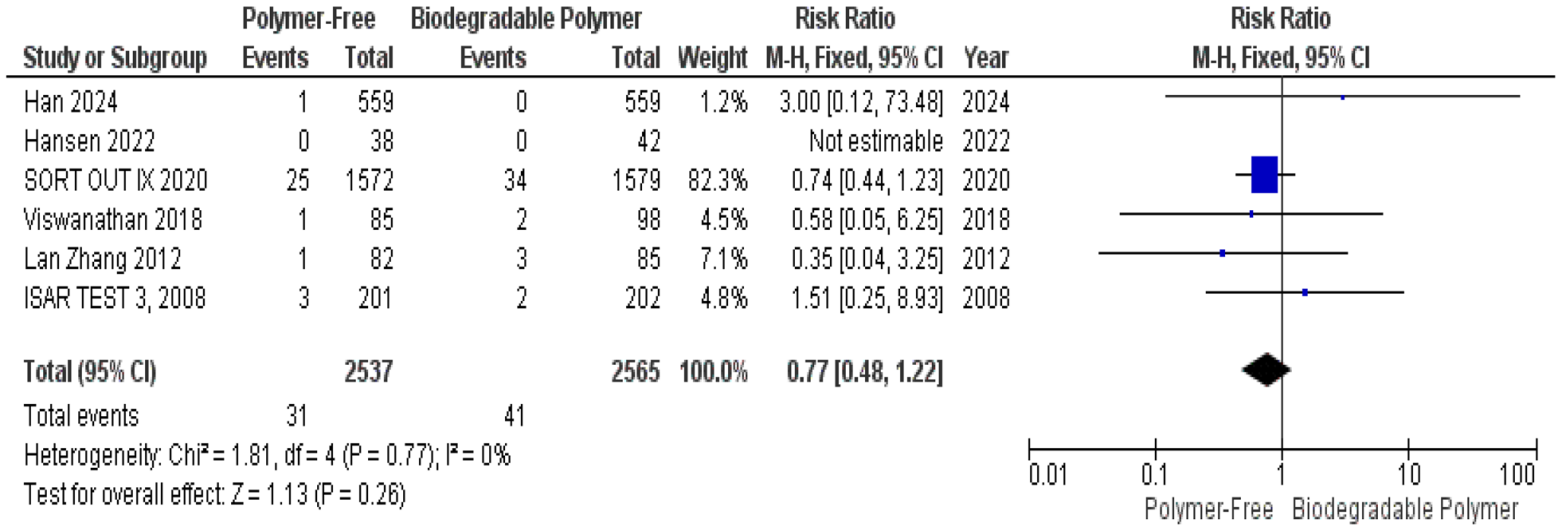
Overall stent thrombosis at 12 months

**Fig. 16:**
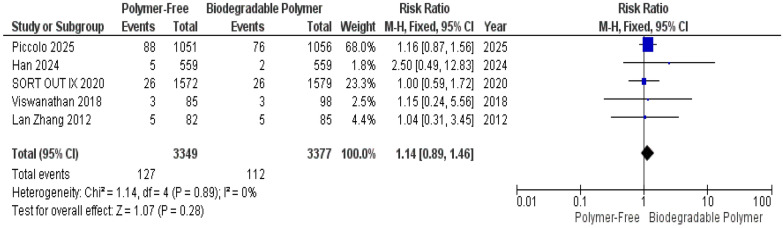
Target lesion-related myocardial infarction (TLR–MI) at 12 months

At 1-year follow-up, no significant differences were observed between the PF–DES and BP–DES groups regarding all-cause mortality (RR = 1.04, 95% CI 0.75–1.44, *P* = 0.82) (Fig. 14), target lesion-related myocardial infarction (TLR–MI) (RR = 1.14, 95% CI 0.89–1.46, *P* = 0.28) (Fig. 15) and overall stent thrombosis (RR = 0.77, 95% CI 0.48–1.22, *P* = 0.26) (Fig. 16). Similarly, these outcomes showed no significant heterogeneity; all-cause mortality (*P* = 0.57, I^2^ = 0%) (Fig. 14), target lesion-related myocardial infarction (*P* = 0.89, I^2^ = 0%) (Fig. 15) and overall stent thrombosis (*P* = 0.77, I^2^ = 0%) (Fig. 16).

**Fig. 17:**
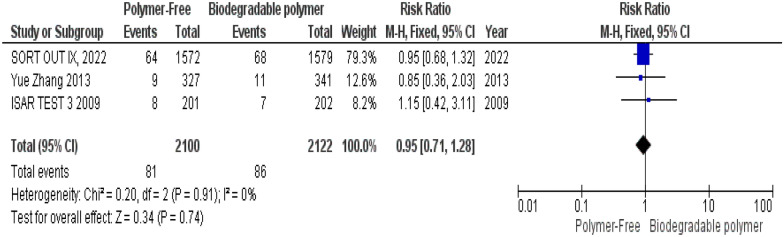
All-cause mortality at 24 months

**Fig. 18:**
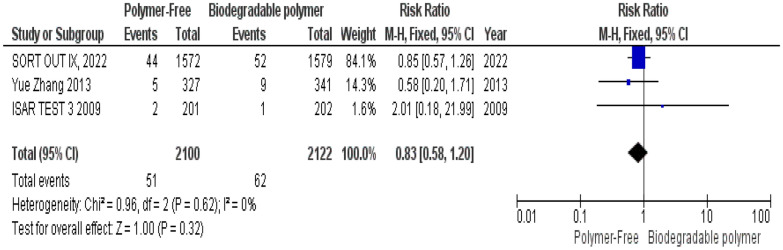
Overall stent thrombosis at 24 months

**Fig. 19:**
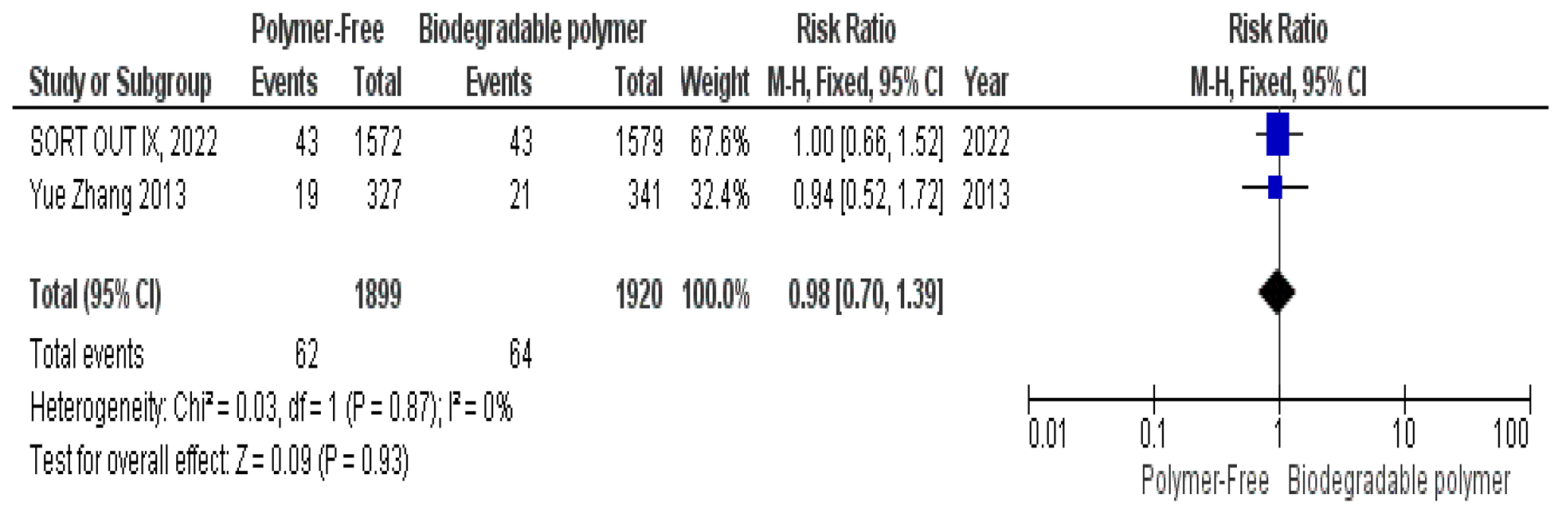
Target lesion-related myocardial infarction (TLR–MI) at 24 months

At 2-year follow-up, no statistically significant differences were observed between both groups as well regarding all-cause mortality (RR = 0.95, 95% CI 0.71–1.28, *P* = 0.74) (Fig. 17), overall stent thrombosis (RR = 0.83, 95% CI 0.58–1.20, *P* = 0.32) (Fig. 18) and target lesion-related myocardial infarction (TLR–MI) (RR = 0.98, 95% CI 0.70–1.39, *P* = 0.93) (Fig. 19). Such outcomes showed no statistically significant heterogeneity in their analysis either; all-cause mortality (*P* = 0.91, I^2^ = 0%) (Fig. 17), overall stent thrombosis (*P* = 0.62, I^2^ = 0%) (Fig. 18) and target lesion-related myocardial infarction (TLR–MI) (*P* = 0.87, I^2^ = 0%) (Fig. 19).

### Angiographic outcomes at 6–8 months

**Fig. 20:**
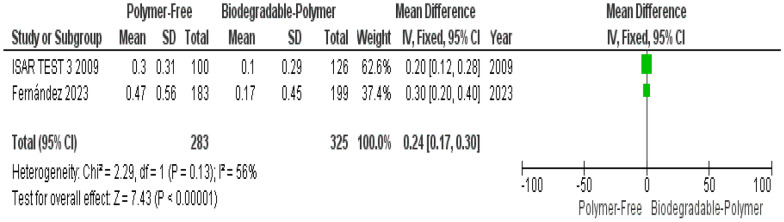
In stent late lumen loss at 6–8 months

At 6–8-month follow-up, angiographic outcomes showed that in-stent late lumen loss (LLL) was significantly higher with PF–DES compared to BP–DES (mean difference [MD]: 0.24 mm, 95% CI 0.17–0.30, *P* < 0.00001), with moderate but statistically non-significant heterogeneity (I^2^ = 56%, *P* = 0.13) (Fig. 20).

**Fig. 21:**
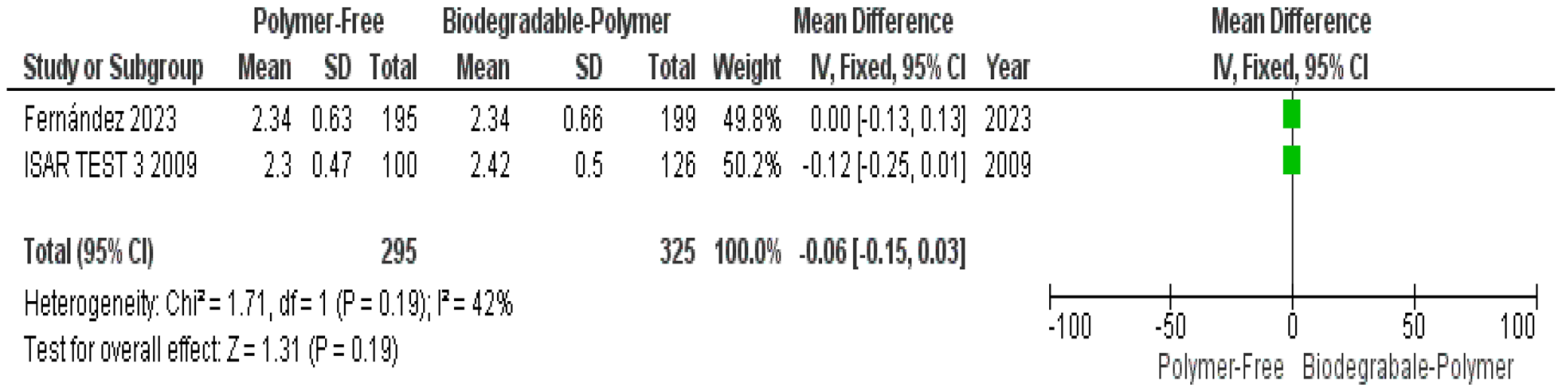
Minimal lumen diameter at 6–8 months

Minimal lumen diameter (MLD) did not differ significantly between groups (MD: –0.06 mm, 95% CI –0.15 to 0.03, *P* = 0.19), and heterogeneity was low and not statistically significant (I^2^ = 42%, *P* = 0.19) (Fig. 21).

## Discussion

In this systematic review and meta-analysis of ten RCTs involving 9,020 patients, we compared the clinical safety, efficacy, vascular healing characteristics and angiographic findings of polymer-free drug-eluting stents (PF–DES) and biodegradable-polymer drug-eluting stents (BP–DES) in patients undergoing PCI. PF–DES were associated with higher rates of target lesion revascularization (TLR) at both 1-year and 2-year follow-up, whereas no significant differences were found in target vessel revascularization (TVR), definite or overall stent thrombosis, target lesion-related myocardial infarction, all-cause death, or cardiac death or in other optical coherence tomography (OCT)-derived markers of vascular healing. In addition, PF–DES was associated with significantly greater in-stent late lumen loss at 6–8 months, whereas minimal lumen diameter remained comparable between groups.

The most notable finding of this analysis was the increased rate of TLR in the PF–DES group, suggesting reduced lesion-level efficacy compared with BP–DES. This result was consistent across the largest contributing studies, including Gregersen et al. [12] and Piccolo et al. [22]. The absence of a corresponding difference in TVR suggests that this efficacy signal is localized to the stented segment rather than the target vessel as a whole.

The concomitant observation of greater in-stent late lumen loss at 6–8 months provides a plausible angiographic correlation for the increased rates of TLR. Late lumen loss reflects the extent of neointimal hyperplasia following stent implantation and has been shown to correlate with subsequent target lesion revascularization in contemporary drug eluting stents. In particular, a meta-analysis by Asano et al. demonstrated a strong relationship between in-stent late lumen loss and clinically driven TLR across DES trials [24]. These findings may reflect variability in drug-delivery kinetics in PF–DES, which rely on alternative release mechanisms, such as surface coatings or microporous reservoirs, rather than controlled polymer-based drug elution. Furthermore, the absence of a polymer-based carrier too may result in earlier drug elution [25], potentially limiting sustained suppression of neointimal hyperplasia during the healing phase compared to biodegradable polymer platforms, which provides a more sustained release of antiproliferative agents as the polymer degrades over weeks to months.

Regarding safety endpoints, both stent platforms demonstrated comparable outcomes at both 1- and 2-year follow-up periods. Cardiac death occurred at similar rates, and definite stent thrombosis showed no significant difference. Overall stent thrombosis, encompassing definite and probable events, also showed no significant difference. These results align with prior studies, such as Yue Zhang et al. [20] and Viswanathan et al. [21], which found no major safety concerns with polymer-free designs in stable or low-risk populations.

Moreover, all-cause mortality and target lesion-related myocardial infarction were similar between the two groups, further supporting the conclusion that PF–DES, despite higher TLR, do not compromise overall clinical safety at both 1- and 2-year follow-up.

Our findings align with the evidence from individual trials and prior analyses comparing polymer-free and biodegradable-polymer stents. Most of the randomized controlled trials (RCTs) included in this review concluded that PF–DES were non-inferior to polymer-containing DES in terms of clinical outcomes, which is consistent with our pooled results. For instance, the ISAR-TEST-3 trial, one of the earlier studies, reported no significant differences in composite endpoints between PF–DES and BP–DES, although it noted numerically higher TLR rates in the polymer-free arms [16, 26]. At 1-year follow-up in the SORT-OUT IX trial the PF–DES did not meet non-inferiority for target lesion failure compared to BP–DES, driven largely by an increased incidence of TLR with the polymer-free stent [27]. By 2 years, SORT-OUT IX confirmed an approximately twofold higher TLR rate with PF–DES (5.1% vs. 2.6%), while safety outcomes remain comparable between groups [12].

This pattern mirrors our meta-analysis outcome showing higher TLR with PF–DES but no safety disadvantage. Other trials, such as Yue Zhang et al. 2012, Lan Zhang 2013, Viswanathan 2018, although generally smaller and individually underpowered to detect small differences, typically found no significant outcome differences at 1 year [20, 21, 23].

Notably, no study has demonstrated any increase in stent thrombosis with polymer-free stents. If anything, the expectation was that eliminating polymers might reduce late thrombotic risk. SORT-OUT IX reported no significant difference in definite stent thrombosis at 2 years (0.7% PF–DES vs. 0.5% BP–DES) [12], and several similar trials likewise had comparable stent thrombosis incidences, reporting zero or near-zero events in both arms. In our analysis, stent thrombosis rates were low and did not differ between PF–DES and BP–DES, corroborating the safety findings from individual RCTs including those with long term follow-up [22, 28]. These concordant findings support the conclusion that polymer strategy does not significantly influence safety outcomes in the contemporary DES era.

Recent meta-analyses and network comparisons in the field similarly report no major outcome differences among modern DES designs, supporting the concept that the elimination of durable polymer has not led to significant superiority or inferiority in clinical endpoints [29, 30].

A key focus of this review was the comparison of vascular healing between PF–DES and BP–DES as assessed by OCT, which provides essential insights into early vascular healing and stent integration. In our meta-analysis, three studies evaluated uncovered struts, neointimal thickness, and malapposed struts. No significant differences were found between PF–DES and BP–DES in uncovered struts or neointimal thickness both characterized by substantial heterogeneity (I^2^ = 98% and 99%, respectively), which was not explained by leave-one-out analyses. Given the limited number of studies reporting these findings and the variability in imaging timepoints, these OCT findings should be interpreted as exploratory and hypothesis-generating.

For malapposed struts, the initial pooled estimate including all studies reporting this outcome was non-significant and showed significant heterogeneity. Although exclusion of a heterogeneity-driving study resulted in a statistically significant pooled estimate with no residual heterogeneity, this finding was dependent on study inclusion and should, therefore, be interpreted with caution. Therefore, the primary analysis including all studies is emphasized, which does not indicate a consistent difference in malapposition between stent types.

Our OCT-related findings are in line with the findings from individual RCTs. Fernández et al. reported a high percentage of uncovered struts at 1 month across different stent types, with no significant differences between polymer-free, biodegradable-polymer, and durable-polymer DES [14]. Hansen et al. observed similar strut coverage in both groups [13]. Irurueta et al. in the FRIENDLY-OCT study also reported no significant differences in strut-level healing, despite different drug formulations and release kinetics [15]. Similarly, Fernández-Rodríguez et al. showed numerically higher uncovered struts in the BP–DES group, though this was not statistically significant [14].

With respect to neointimal thickness, Hansen et al. reported comparable neointimal hyperplasia between polymer-free and biodegradable-polymer stents at 1 month in patients with STEMI [13], consistent with the non-significant difference in neointimal thickness observed in our pooled analysis. In contrast, Irurueta et al. reported better overall strut coverage with a novel polymer-free stent at 3 months [15]. This difference may be explained by variation in OCT imaging timepoints, or specific stent designs and platform characteristics. Overall, findings from the individual studies are consistent in showing no clear differences in early strut coverage or neointimal thickness between stent types while highlighting the heterogeneity and exploratory nature of OCT-derived findings.

In addition to clinical and OCT-based endpoints, angiographic outcomes at 6–8 months were reported in two randomized controlled trials: ISAR-TEST-3 [16] and Fernández et al. [14]. Both studies evaluated in-stent late lumen loss (LLL) and minimal lumen diameter (MLD) as markers of restenosis. In ISAR-TEST-3, PF–DES demonstrated lower LLL than BP–DES (0.30 mm vs. 0.10–0.14 mm), whereas Fernández et al. found a slightly higher LLL with PF–DES (0.19 mm vs. 0.20–0.28 mm). Despite this variation, the pooled analysis revealed a statistically significant increase in LLL associated with PF–DES, indicating greater neointimal growth compared to BP–DES. For MLD, both trials reported similar values across stent types, with the meta-analysis confirming no significant difference. These results suggest that although lumen dimensions remain similar, biodegradable-polymer DES may offer improved mid-term efficacy in suppressing restenosis, as reflected by lower late lumen loss.

Overall, the findings of this meta-analysis indicate that both PF–DES and BP–DES demonstrate comparable safety profiles, consistent with the concept that modern stent performance is increasingly driven by platform- and device-specific characteristics rather than polymer strategy alone. In this context, the absence of consistent differences in most clinical and OCT-derived outcomes underscores the importance of interpreting observed associations within the limitations imposed by heterogenous device designs and follow-up protocols.

From a clinical perspective, the higher rates of target lesion revascularization observed with PF–DES in pooled analyses warrant consideration, particularly in patients at increased risk of restenosis, such as those with diabetes or complex coronary anatomy. However, given the heterogeneity of included platforms and the exploratory nature of OCT findings, these results should not be interpreted consistently across all polymer-free stent platform designs. Instead, they highlight the need for individualized device selection based on patient risk profile and lesion characteristics.

Future randomized controlled trials with longer follow-up periods and standardized imaging protocols are needed to clarify the long-term performance of PF–DES, especially in high-risk subgroups, such as patients with diabetes, chronic kidney disease, or those with prior stent failure. In particular, head-to-head trials using uniform imaging protocols (e.g., same OCT timing, outcome definition for healing indices) may help clarify whether specific device designs or drug-elution strategies offer meaningful advantages in selected patient populations. Moreover, studies investigating drug elution kinetics, and stent interaction with vascular tissue would help guide future refinements in PF–DES design to optimize efficacy without compromising safety.

### Limitations

This meta-analysis has several limitations that should be interpreted within the context of distinct sources of heterogeneity. First, important statistical heterogeneity was observed across OCT-derived outcomes. While most clinical outcomes (TVR, MI, mortality) demonstrated low statistical heterogeneity, OCT-derived outcomes showed extreme heterogeneity for uncovered struts I^2^ = 98% and neointimal thickness struts I^2^ = 99% which was not resolved by sensitivity analysis, as well as substantial heterogeneity for malapposed struts, which was partially reduced by sensitivity analysis. Although random-effects models were applied to account for between-study variance, and forest plots were inspected for outlier influence, statistical adjustment alone does not resolve underlying clinical and methodological non-comparability.

These findings likely reflect the second type of heterogeneity which is the imaging heterogeneity inherent to the included OCT studies. OCT follow-up was performed at different timepoints ranging from 1 to 3 months and imaging protocols varied across trials. Given the dynamic nature of vascular healing, early and later OCT assessments may capture distinct biological phases of strut coverage and neointimal formation, therefore, limiting direct comparability. Additional variability in strut thickness (ranging from 70 to 100 µm) and device-specific healing profiles further contribute to inconsistency across OCT outcomes. Accordingly, OCT findings should be interpreted with caution and considered exploratory and hypothesis-generating.

Nonetheless, substantial clinical heterogeneity existed across included trials. Differences in stent platforms, antiproliferative drugs (sirolimus, biolimus and amphilimus), device-specific design features and procedural characteristics may influence vascular healing and restenosis independently of polymer strategy limiting the ability to draw solid conclusions. In addition, endpoint definitions were extracted as reported in the original trials, and minor variations in definitions may have contributed to clinical heterogeneity.

Furthermore, several outcomes, particularly OCT findings and angiographic measures, were based on a limited number of trials and events, reducing statistical power and precision of the effect estimates and increasing susceptibility to small-study effects.

Despite these limitations, our meta-analysis is strengthened by the inclusion of only randomized controlled trials, which minimizes selection bias, and by the large, pooled sample size of 9,020 patients, enhancing statistical power and the generalizability of the findings.

## Conclusions

In this systematic review and meta-analysis of randomized controlled trials, polymer-free drug-eluting stents were associated with higher rates of target lesion revascularization and greater in-stent late lumen loss compared to biodegradable-polymer drug-eluting stents, while no differences were observed in major safety outcomes. OCT-derived findings were heterogenous and should be interpreted cautiously. These results support an individualized and device-specific approach to stent selection and highlight the need for further randomized studies with standardized imaging and longer follow-up periods.

## Data Availability

All data generated or analyzed during this study are included in this published article and its supplementary information files.
